# Acesso e aguardo pela estomizaáo segundo pessoas com cáncer colorretal: estudo etnográfico

**DOI:** 10.15649/cuidarte.1175

**Published:** 2023-03-29

**Authors:** Antonio Jorge Silva, Thaís Cristina Flexa Souza, Mary Elizabeth de Santana, Helena Megumi Sonobe, Ingrid Magali de Souza Pimentel, Jacira Nunes Carvalho

**Affiliations:** 1 . Universidade de Sáo Paulo, Escola de Enfermagem de Ribeiráo Preto - USP. Ribeiráo Preto, Sáo Paulo, Brasil. Email: juniorjorge 94@hotmail.com Universidade de São Paulo Universidade de Sáo Paulo Brazil juniorjorge 94@hotmail.com; 2 . Universidade Federal do Pará - UFPA. Belém, Pará, Brasil. Email: thaisflexxa@gmail.com Universidade Federal do Pará Universidade Federal do Pará Brazil thaisflexxa@gmail.com; 3 . Universidade Federal do Pará - UFPA. Belém, Pará, Brasil. Email: marybete@ufpa.br Universidade Federal do Pará Universidade Federal do Pará Brazil marybete@ufpa.br; 4 . Universidade de Sáo Paulo, Escola de Enfermagem de Ribeiráo Preto - USP. Ribeiráo Preto, Sáo Paulo, Brasil. Email: megumi@eerp.usp.br Universidade de São Paulo Universidade de Sáo Paulo Brazil megumi@eerp.usp.br; 5 . Universidade Federal do Pará - UFPA. Belém, Pará, Brasil. Email: imbarleta@yahoo.com.br Universidade Federal do Pará Universidade Federal do Pará Brazil imbarleta@yahoo.com.br; 6 . Universidade Federal do Pará - UFPA. Belém, Pará, Brasil. Email: jacirancarvalho@gmail.com Universidade Federal do Pará Universidade Federal do Pará Brazil jacirancarvalho@gmail.com

**Keywords:** Neoplasias Colorretais, Acesso aos Servidos de Saúde, Sistema Único de Saúde, Medicalizaáo, Sociologia Médica., Colorectal Neoplasms, Health Services Accessibility, Unified Health System, Medicalization, Sociology, Medical., Neoplasias Colorrectales, Accesibilidad a los Servicios de Salud, Sistema Único de Salud, Medicalización, Sociología Médica.

## Abstract

**Objetivo:**

Conhecer o acesso e aguardo pela estomizaáo de _ pessoas adoecidas por cáncer colorretal no nível terciário do Sistema Único de Saúde.

**Materiais e Métodos:**

Estudo etnográfico fundamentado na Sociologia da Saúde, com 8 familiares e 14 adoecidos em Centro de Alta Complexidade em Oncologia, Brasil. Os dados foram coletados entre outubro de 2018 a maro de 2019, com observado participante e náo participante, registro em diário de campo e entrevista semiestruturada. Os depoimentos e notas etnográficas após triangulado foram submetidos a análise indutiva de conteúdo em seis etapas.

**Resultados:**

Apreenderam- se "A história do adoecimento entrecortada pelas dificuldades” e "As perdas no processo de paciéncia-resiliéncia no percurso”.

**Discussáo:**

Em busca de validades biomédicas foram reportados tres tipos de acesso ao sistema médico, assim como os subsentidos "paguei no particular” e atraso diagnóstico mobilizaram via oficial e náo oficial no Sistema Único de Saúde. O capital social foi analisado como premente no adoecimento, uma rede de contatos sem a qual o acesso dos usuários é impactado. No nivel terciário enquanto aguardam pela estomizaáo desvelou-se a resiliéncia náo como recurso heroico, mas como recurso pessoal e coletivo diante do percurso dificultoso e da semi-reclusáo na instituido total onde vivenciam medos, fadiga e dores.

**Conclusáo:**

O acesso contou com mobilizado relacional e uma gama de vias até a internado com parte do percurso na saúde suplementar, já o aguardo pela estomizaáo mitiga a paciéncia ao passo que torna a resiliéncia um recurso benéfico na espera pela programado cirúrgica.

## Abstract (en)

**Introduction:**

The access of people with colorectal cancer to surgical treatment with stomization implies a difficult itinerary through the network of care, and when accessing the tertiary level in an apprehensive preoperative waiting.

**Objective:**

To know the access and waiting for stomization of people sick with colorectal cancer at the tertiary level of the Brazilian Unified Health System.

**Materials and Methods:**

Ethnographic study based on the Sociology of Health, with 8 relatives and 14 patients in a High Complexity Oncology Center, Brazil. Data were collected between October 2018 to March 2019, with participant and non-participant observation, field diary recording, and semi-structured interview. Statements and ethnographic notes after triangulation were subjected to inductive content analysis in six steps.

**Results:**

"The history of illness interspersed with difficulties" and "The losses in the process of patience-resilience along the way" were apprehended.

**Discussion:**

Three types of access were reported, as well as the sub meanings "I paid privately" and the diagnostic delay mobilized by official and unofficial means in the Unified Health System. Social capital is pressing, a network without which users' access is impacted. At the tertiary level while waiting for stomization, resilience was unveiled not as a heroic resource, but as a personal and collective resource.

**Conclusion:**

The access counted on relational mobilization and a range of paths to hospitalization with part of the journey in supplementary health, while the waiting for stomization mitigates patience making resilience a beneficial resource in the wait for surgical programming.

## Resumo

**Introducción:**

El acceso de las personas con cáncer colorrectal al tratamiento quirúrgico con estomización implica un difícil itinerario a través de la red asistencial, y al acceder al nivel terciario en una aprensiva espera preoperatoria.

**Objetivo:**

Conocer el acceso y el aguante de las personas afectadas por el cáncer colorrectal en el nivel terciario del Sistema Único de Salud.

**Materiales y Métodos:**

Estudio etnográfico basado en la Sociología de la Salud, con 8 familiares y 14 pacientes en Centro Oncológico de Alta Complejidad, Brasil. Los datos se recogieron entre octubre de 2018 y marzo de 2019, con observación participante y no participante, registro de diario de campo y entrevista semiestructurada. Los testimonios y las notas etnográficas, tras la triangulación, se sometieron a un análisis de contenido inductivo en seis etapas.

**Resultados:**

Se aprende "La historia del adoctrinamiento entrecortado por las dificultades" y "Las pérdidas en el proceso de pacificación- resiliencia en el curso".

**Discusión:**

Se reportaron tres tipos de acceso, así como los subsentidos "pagué en el privado" y el atraso diagnóstico se movilizaron vía oficial y no oficial en el Sistema Único de Salud. El capital social es apremiante, una red de contactos sin la cual el acceso de los usuarios se ve afectado. En el nivel terciario, mientras aguantan por la estomatología, la resistencia se revela no como un recurso heroico, sino como un recurso personal y colectivo.

**Conclusión:**

El acceso a la movilización relacional y a una gama de vías hasta la internación con parte del curso en la salud suplementaria, ya que la espera por la estomización mitiga la pacificación haciendo de la resiliencia un recurso benéfico en la espera por el programa quirúrgico.

## Introdujo

A política de atengáo oncológica brasileira busca garantir o acesso a servidos especializados e de alta complexidade, estabelecendo critérios para os servidos desta natureza no ámbito do Sistema Único de Saúde (SUS) intimamente ligados ao modelo de atengáo a saúde que passou a ser estruturado a partir de 2011. Entretanto, sobreleva-se que a fragilidade desta integrado culminando em um acesso demorado ao nível terciário^1^.

A conhecida demora implica em um aumento significativo, especialmente entre os homens, da incidencia de cáncer colorretal (CCR) e por conseguinte da mortalidade, como já está sendo documentado nas regióes Norte e Nordeste do Brasil, sabendo que nacionalmente estima-se para cada um dos anos de 2020, 2021 e 2022 a frequencia anual de 20.520 casos de cáncer de cólon e reto em homens e 20.470 em mulheres^2^^,^^3^. Por conseguinte a descoberta dos sintomas é elemento crucial^4^^,^^5^, e a persistencia dos mesmos conclama que o diagnóstico e encaminhamento sejam depreendidos.

Neste bojo, sáo sintomas importantes a dor abdominal, hematoquezia, melena, dor nas costas, perda de peso, urgencia fecal, constipado e/ou diarreia, contando com períodos prolongados até validarem- nos com médicos, que fazem com que usuários os esquegam ou minimizem-nos caso diminuam de intensidade e frequencia. Assim, pessoas com CCR necessitaráo de exames confirmatórios como colonoscopia, consultas referenciadas devido as medicagóes prescritas, um acompanhante familiar ou náo, alimentado, moradia próxima ao tratamento e frequentemente vivenciam situagóes domiciliares difíceis^6^^,^^7^ devido a consequente mobilizagáo de capacidades financeiras e mecanismos sociais.

Portanto, acessar os níveis de atengáo para tratamento cirúrgico para tumores sólidos e confecgáo de estomia, depende tanto da postura do adoecido oncológico, das Centrais de Regulagáo e peculiaridades da regiáo de saúde na qual está inserido com residencia fixa ou náo. O itinerário comega em Unidades Básicas de Saúde (UBS) ou na atengáo de média complexidade, para entáo o usuário ser regulado via SUS para a alta complexidade, evidentemente a exiguidade de servigos desta natureza faz com que usuários com cáncer no do trato digestivo se desloquem para regióes metropolitanas ou cidades de grande porte a fim de efetuar o tratamento. Na oncologia as interfaces entre os níveis no SUS se dáo em fluxos para regióes com a maior concentragáo de servigos de alta complexidade, de forma piramidal, porém mesmo assim os usuários tragam fluxos náo oficiais de acesso^7^.

Desta maneira, ouvir acerca do percurso de pessoas narrando transigóes na rede em um servigo de alta complexidade no qual aguardam a cirurgia com estomizagáo, permite compreender o acesso ao tratamento e ainda as experiencias produzidas na internagáo. Considera-se o lugar de escuta no nível terciário de atengáo como valioso, pois a rede de relagóes das pessoas que buscam atendimento e tratamento no meio urbano é densa e sáo raríssimos os casos de pessoas que perdurem sem elas^8^. Estudos com a fundamentagáo sociológica exploram a tendencia de humanizagáo, subsistemas e itinerário até o sistema profissional-biomédico^4^^,^^5^.

Consequentemente, sáo objetos da Sociologia da Saúde a explicagáo da mobilizagáo de capacidades após o adoecimento, territorializagáo física ou social e experiencia corporal^9^. Alguns de seus conceitos constituintes sáo: medicalizagáo com a constante de validagóes médicas e náo médicas do adoecimento^10^, institucionalizagáo cultural e “instituigóes totais” defendidas por Erving Goffman^11^ como aqueles locais de internagáo passageira ou náo (hospitais psiquiátricos, asilos, prisóes e conventos), reclusáo ou semi-reclusáo com: autoridade, rotinas agendadas, equipe dirigente e redes de resiliencia coletivas.

Seguindo o exposto o problema de investigagáo foi: Quais as narrativas de acesso e de aguardo pela estomizagáo de pessoas adoecidas por cáncer colorretal no nível terciário? E diante disto, o objetivo foi conhecer o acesso e aguardo pela estomizagáo de pessoas adoecidas por cancer colorretal no nível terciário do Sistema Único de Saúde.

## Materiais e Métodos

Estudo etnográfico assentado no paradigma compreensivo-interpretativo da Sociologia da Saúde. A Sociología é interativa gerando impactos na política de saúde, humanizado de servidos e descortina experiencias, prevé a conexáo reflexiva dos conhecimentos da biologia, humanas e educado. Transcende-se a explicado apenas da doenga e averíguam-se os sentidos (sentimentos), considerando que as pessoas dispóem de relagóes materiais (naturais e culturais) considerando múltiplas vinculagóes do contexto de adoecimento^9^^,^^12^. Neste contexto, um protocolo de pesquisa etnográfica foi pensado a partir do Consolidated criteria for reporting qualitative research (COREQ)^13^, elaborado por pesquisadores com experiéncia em pesquisa qualitativa e publicagóes na área, destacando-se no tocante ao “Relacionamento com participantes” o tempo de interagáo de 5 dias até no máximo 1 més de contato com os internados no aguardo da programagáo cirúrgica, e contato único para aqueles ambulatoriais.

Nas relagóes de usuário-familiar-equipe existem agentes de poder simbólico estrutural (dominados- dominantes) expressos na rede de atengáo, comportamentos alicergados por padróes de poder nas instituigóes expressos durante a internagáo, equivalendo portanto a um campo profícuo para estudar valores do senso comum, aliangas-rupturas institucionais, lutas, paradigmas de gestáo e ainda de violéncia simbólica perpetrada pelo sistema^9^^,^^12^. Para abranger singularidades vivenciadas no processo de ver-se estomizado após uma transigáo de níveis de atengáo, a sociologia focaliza a contextura de indivíduos dependentes uns dos outros subsistindo pela unidade de suas fungóes sendo a ciéncia das fungóes societárias^8^.

A coleta desenvolveu-se em dois cenários de Centro de Alta Complexidade em Oncologia (CACON) na regiáo metropolitana de Belém, estado do Pará, Brasil, na clínica cirúrgica oncológica abdominal e ambulatório antineoplásico. Assevera-se que além da oncologia o CACON comporta dezessete especialidades, entre elas neurocirurgia, transplantes de córnea e rins. A instituigáo é uma autarquia e hospital de ensino referéncia em servigos médico-hospitalares de alta complexidade para a populagáo oriunda da regiáo Norte do Brasil e, incluso algumas cidades do Nordeste, o acesso oficial a mesma dá- se após referéncia dada a confirmagáo diagnóstica e a internagáo por meio dos Sistemas de Regulagáo (SISREG) e Sistema Estadual de Regulagáo (SER).

Logo, a selegáo de participantes primários foi de adoecidos pelo CCR em tratamento cirúrgico com estomizagáo, residentes temporariamente ou permanentemente na regiáo metropolitana de Belém e maiores de 18 anos, sendo o maior Índice de Desenvolvimento Humano dos municípios de procedéncia dos depoentes de 0,768 (Sáo Luís no estado do Maranháo) e o menor 0,515 (Viseu no estado do Pará) na consulta ao Atlas do Desenvolvimento Humano no Brasil com dados do último censo populacional^14^; excluíram-se os que regressaram sem estomizagáo do centro cirúrgico devido ao protocolo de pesquisa contemplar o aguardo e retorno com estomizagáo para emersáo de sentidos específicos. Desta forma considerou-se o binomio paciente-familiar após um trabalho de campo de dois meses, pois dificuldades de coleta foram apreendidas e a inclusáo do familiar como participante secundário consistia como um benefício após anuéncia do adoecido. Foram fontes de dados 22 pessoas, esclarecendo: entrevistas e contatos com oito binomios adoecido-familiar (16 pessoas) e com seis adoecidos respondentes sem a necessidade de ajuda.

Seguindo o protocolo obedeceu-se: descrigáo em diário de campo de eventos de uma fase inicial até um desfecho; registro das interpretagóes em notas etnográficas operacionais, metodológicas e teóricas - compostas pela data e lista de participantes renovada com admissóes e altas e eventos observados (palavras-chave, descrigáo cronológica, interpretagóes e apontamentos para o próximo encontro). Sendo registradas imediatamente após a saída da enfermaria no posto de enfermagem. As técnicas empregadas foram observado participante, náo participante e a entrevista semiestruturada do tipo interpretativa^15^, a observado participante atendeu a descrigáo ordenada e solicitado de narrativas.

As etapas da coleta de dados desenvolvida pelo primeiro autor (enfermeiro), perduraram de outubro de 2018 a margo de 2019 com: 1) aproximagáo dos binomios na admissáo, registrando o semblante, conhecimento sobre a doenga e experiencia náo verbal nas enfermarias. Somente no trabalho campo foi especificado o que poderia ser apreendido, registrado e replicado; 2) observagáo náo participante na Clínica Cirúrgica e no ambulatório para identificar a dinámica, organizagáo e rotina (rituais); 3) observagáo participante, entrevista em profundidade próxima a alta da clínica e uso de diário de campo anotando informagóes relevantes, nexos entre as observagóes e conceptualizagóes em notas.

Ao final durante o pós-operatório as perguntas sobre determinantes do acesso e do aguardo na internagáo, levando em conta a produgáo de dados anterior a qual engajou observagáo centrada no olhar e em todos os sentidos, foram: Como conseguiu a internagáo?; O que este hospital significa para voce?; O que lhe ajuda a permanecer aqui?; Tem alguma dificuldade aqui?; Quais profissionais tem vindo lhe orientar?; Como o senhor(a) tem sido tratado(a)?. Estas comegaram a ser aplicadas sempre ao final da produgáo de dados e após um teste piloto de uma semana direcionado aos usuários com CCR admitidos no CACON, no início da insergáo do pesquisador no cenário, as questóes foram pensadas unicamente para o nível de atengáo terciária.

Tais entrevistas foram áudio-gravadas e convertidas em formato MP3. A triangulagáo aconteceu entre notas etnográficas e entrevistas transcritas na íntegra sendo procedimento indispensável além do retorno do material transcrito para os participantes oferecerem feedbacks. Assomaram-se também dados obtidos na clínica cirúrgica e no ambulatório antineoplásico: 1) notas referentes ao pré- operatório trianguladas subsequentemente a entrevista semiestruturada no pós-operatório gerando um arquivo Microsoft Word para cada paciente e/ou binomio; 2) notas dos abordados e observados no pré-operatório, porém náo entrevistados, foram agrupadas em arquivo Microsoft Word, amparando conceptualizagóes. Em conformidade com a ciencia aberta, os dados das entrevistas usados para o relatório geral preservando a confidencialidade estáo disponíveis para consulta pública em Data-set^16^.

A análise de similitudes e de distingáo foi depreendida com codificagáo de temas em um arquivo matriz nas etapas: 1) familiarizagáo com os dados - a etnografia permitiu ao pesquisador explorar os pontos críticos das entrevistas transcritas, com solicitagáo de novas narrativas com o decorrer dos contatos; 2) geragáo de categorias - codificagáo sistemática de características concretas dos fatos também amparada pelo diário de campo; 3) geragáo de unidades para comportar as categorias por proximidade temática; 4) interpretagáo; 5) nomes para os temas das categorias; 6) relatório^17^. O relatório foi apresentado na dissertagáo de mestrado “Os sentidos do adoecimento pelo cáncer colorretal: estudo etnográfico”, esta pesquisa é oriunda da unidade “Os sentidos do acesso”.

Como cuidado ético empregou-se o código alfanumérico P1-P11 para pacientes, PA12-PA13 para pacientes ambulatoriais, F1-F7 para familiares e FA8 para familiar do ambulatório. Todos os participantes assinaram o Termo de Consentimento Livre Esclarecido. A coleta depreendeu-se após a aprovagáo por dois Comites de Ética em Pesquisa da Universidade Federal do Pará e da instituigáo, respectivamente Certificado de Apresentagáo para Apreciagáo Ética (CAAE) 90503818.8.0000.0018 e CAAE 90503818.8.3001.5550.

## Resultados

A amostra foi de quatorze adoecidos e oito familiares. Apenas relagáo aos adoecidos, sete homens e sete mulheres com ocupagóes como: aposentados, donas de casa, agricultores, um mecánico, uma auxiliar de servigos gerais, um garimpeiro e uma freira, suas procedencias: Belém, Barcarena, Canaá dos Carajás, Capanema, Igarapé-Agu, Igarapé-Miri, Máe do Rio, Maracaná, Viseu, Parnaíba no Piauí e Sáo Luís (cidade do nordeste brasileiro no estado do Maranháo). A totalidade dispunha somente do SUS como sistema de cuidado a saúde, do diagnóstico até a internagáo o período de tempo decorrido foi: menos de 3 meses para quatro participantes (28,56%), entre 3 meses-1 ano para cinco (35,70%), entre 1 ano-2 anos para um participante (7,14%) e 2 anos ou mais para 4 participantes (28,56%).

Durante o percurso na clínica a coleta de dados foi marcada pela ausencia de estomizagáo devido a anastomose no bloco cirúrgico, estadiamento avangado sem indicagóes terapeuticas, reabordagens e óbito no Centro de Terapia Intensiva. Com o processo de análise da unidade “Os sentidos do acesso” as categorias de sentidos a seguir foram elaboradas.

A história do adoecimento entrecortada pelas dificuldades.

**Figura 1:**
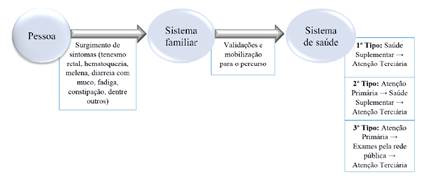
Percursos para o acesso até o aguardo pela estomizaáo na atenfáo terciária

A anormalidade é detectada e o percurso iniciado com compartilhamento com sistema de cuidado informal e depois o biomédico. Todo percurso é sintetizado pela Figura 1.

*Apresentou o sangramento [retal]. Comegou com uma prisao de ventre, tinha o intestino regular, de repente evoluiu para uma diarreia líquida branca, sem nada de fezes. Foi se desidratando e esperou para fazer a colo, e foi descoberto que tinha alguma coisa. Aí foi para biópsia e encaminharam. (Síntese F1, F7, FA8)*

*Os outros médicos tratavam como hemorroidas, como eu nao podia fazer nenhum procedimento por causa da gravidez tive de ganhar o bebé. Foram quinze dias depois da cesárea que eu fiz a colonoscopia e com um més fiz a cirurgia. (PA13)*

*“É hemorroidas”, passou um ano e fomos para outro “É hemorroidas”. O terceiro médico eu disse: “Sinto latejar” e fez o toque. Me mandou para outro médico e deu uma secregao no vidro pra eu fazer no laboratório, chegou 10 dias e deu que era um tumor maligno [diário de campo: esses exames foram por onde? ] Foi particular. (PA12)*

Refletem sobre a falta de uma cultura preventiva de cuidados a saúde.

*Eu corria no laboratório fazer o exame particular rápido, pagando achava que já estava curado. Uma vez o técnico me mostrou “Olha tem uma diferenga no teu ultrassom procura um especialista”. Só que eu como nao sentia dor falava “Égua a gente vai no médico tá bonzinho, lá ele arruma um bocado de doenga para gente”. Descobri assim diabetes e pressao alta, passava remédio eu tomava só um pouco, abandonava e ia para manguaga. No outro dia ia curtir a ressaca, meter a mao no bolso vazio. (P1)*

*“Bora no médico ver como tá sua saúde? ” ela dizia “Estou boa”. Quando resolveu ir para hospital chegava e dizia que estava com dor debaixo da costela, aplicavam um remédio para dor na emergéncia, um sorinho. Acabava ela dizia "To boa!” (F4)*

*Via o sangue, nao me incomodei porque nao doía. Um dia as meninas viram no papel e disseram "Vamos atrás de remédio! ”, "Qui! Nao tá me doendo! ”. Em abril do ano retrasado eu fiz o meu trabalho [evacuares fecais], e quando terminou eu fui me limpar e tinha [diário de campo: mensura com a falange distal do dedo indicador]. Nunca tinha ido num hospital, com essa idade toda e é a minha primeira doenga perigosa [diário de campo: de Viseu, 72 anos, nunca realizou Papanicolau ou mamografia]. (P5)*

Durante um trabalho de campo de dois meses, pequeñas narrativas de acesso ganham espado com a dependencia dos discursos médicos.

*[...] pediram a bateria de exames, viram que tinha uma coisa errada e encaminharam. Tinha de ser rápido que a “coisa” já estava bem alarmante, foram todos particulares [diário de campo: médicos voluntários visitam o convento]. É bradicardica, tratou um pouco, fez uma consulta e já se internou esperando a cirurgia [diário de campo: nem chegou a fazer quimioterapia?]. Nao chegou, vai fazer quando ela já tiver bem. (F7)*

*Disse que eu ia fazer radioterapia, quimioterapia, cirurgia e depois radioterapia e quimioterapia de novo. “Eu vou lhe explicar: este é o seu reto [diário de campo: faz gesto de fechar as maos encenando], aqui é o intestino, pense no ánus, o seu problema está aqui. Ele é muito em baixo e a radiando vai destruir o seu ánus. Infelizmente a senhora vai usar a bolsa permanente". (P4)*

*Rapaz o médico me disse que essa quimio mata as células que ficaram, meu tumor era maligno solta umas células para outro órgao. A radio era para matar as dores. (PA12)*

Residentes em outras localidades reclamam do deslocamento e as esposas (F2 e F6) colocam-se como adoecidas.

*Fomos no especialista em oncologia. Já fomos para Castanhal, nós fizemos um bocado de exames de “bioquisia". Foi descoberto. (F2)*

*Era muito difícil [quimioterapia e radioterapia]. Passou mal, estava na casa de um amigo e ia em pé no onibus [diário de campo P7: fazia o trajeto de cerca de duas horas de onde estava instalado até Belém]. Vinha de manha, 13 horas ele fazia a radioterapia e 16 horas fazia a quimioterapia, sentia muito enjoo. (F3)*

*A gente procurou o tratamento, o médico fez a colonoscopia e constatou o tumor, fez a biópsia constatou o cáncer. Conseguimos o tratamento fora de domicílio e viemos para Belém, dentro de seis meses que tínhamos descoberto tudo. Entao foi seis meses lá pro lado de Canaa [dos Carajás], ficamos internados 18 dias para fazer a cirurgia, o médico olhou e viu que o tumor estava grande e se fosse fazer a cirurgia ia ficar com deficiencia e pediu para passar para quimio para reduzir. Tomou quimio dois anos, janeiro eles pediram [documentos] e a gente entregou. Entao sao oito meses da gente brigando. (F6)*

O “paguei no particular” foi subsentido no tocante aos exames e consultas.

*Pagamos um exame 800 reais e eu até pedi "Moga por favor deixa mais barato isso. " aí ela disse “Nao tem como senhora, o médico pediu com contraste. "; Fazer o que né?. (F2)*

*Demora muito Para descobrir fez uns exames particulares. (Síntese F1, F3, F4 e F5)*

*Eu penei muito para pode chegar. Nao fiquei internada, foi fazendo exame, primeiro a gente pagou "unszinhos" até onde o dinheiro deu. O dinheiro terminou... (P5)*

*No posto me disseram: "Isso daí nao é nada sério, deve ser uma hemorroida". Voltei para casa e em nenhum momento eles me ajudaram ou me encaminharam, quando comegou a sangrar paguei tudo particular: consulta e exames. (P6)*

Tensóes nos percursos mobilizam a rede de contatos para o acesso a Rede de Atengáo a Saúde (RAS), como no extenso e marcante depoimento de P4 com mais medo da morosidade que do próprio cáncer.

*Em fevereiro de 2017 tapou minha passagem, parece que tinha uma cabega de crianga para sair e eu estava em viagem. Quando eu cheguei em casa foi saindo, eu fiquei uma manha inteira no banheiro, aquele coco preto saindo. Em fevereiro consegui com meu médico e em maio ele disse "A senhora vai fazer estes exames de sangue e uma colonoscopia. Bote seus documentos e deixe esse encaminhamento na secretaria. " Final de maio e eu com estes sintomas todo dia. No final de junho eu volto lá: "Aínda nao tá teu nome, espera que demora. " Fui em julho para saber e era férias, corri atrás de um exame particular e encontrei de 600 e até de 800. Em agosto: “Dá teu papel de novo, porque no vento foi perdido. Bota xerox dos teus documentos. Eu vou pedir o encaminhamento para o doutor" Liguei para uma prima e contei a situando, ela me arrumou uma consulta popular por 350 e estes sintomas pela parte da manha ou da tarde. Uma lama horrível! Fiz a colonoscopia em setembro e a biópsia, cáncer grau 2 e o doutor disse "Pegue esse encaminhamento. Nao era esse resultado que eu queria lhe dar... A senhora corre ontem, pois esse cáncer é agressivo. Pegue esse papel e deixe na secretaria. " Nao me deu medo da doenga, mas me deu medo que perderam meu papel. Eu ia esperar tudo de novo? (P4)*

Os profissionais recomendaram a via regular de acesso ao CACON.

*Éramos leigos, nao sabíamos. Ela [filha] trouxe a biópsia e conversou com a assistente social, comecaram marcando a consulta e demorou mais dois meses. (F2)*

*Eu vim com as minhas filhas, uma que mora aqui e é mais inteligente e conhece por aí, foi se chegando, se chegando, e achou uma mulher aqui que levou ela e agilizou. (P5)*

*Fez uma consulta num posto de saúde e pediram para fazer os exames, depois o diagnóstico e encaminhamento. (Síntese P2, FA8 e PA12)*

*Primeiro eu participei para a família, fomos no posto e nao tinha [atendimento]. Tivemos de pagar consulta, fazer a limpeza intestinal e depois que eu fiz voltei a evacuar normal, mas as dores permaneciam devido aos tumores. Onde a gente mora [Barcarena], quando acontece uma situacao mais grave, os postos de saúde encaminham para a secretaria de saúde e analisam os casos. (PA13)*

Uma alternativa para agilidade foi a rede de contatos para via de entrada nao oficial.

*Eu com meu filho ligamos para minha prima, ela disse "Vai lá com a K no [nome da instituicao]" Com a amizade ela me colocou para dentro e conseguiu a consulta. (P4)*

*Conheceu uma amiga que era esposa de alguém de dentro, um conhecido, falou como era para ir e deu o caminho das pedras aqui dentro. Veio sozinho e ficava praticamente diariamente dentro deste hospital, se marcavam [ambulatório] ele ficava em pé, vinha cedinho e ficava. Quando desmarcavam ele ia encher o saco. (F3)*

*Conseguimos através de amigos, pois sozinhos nós nao conseguiríamos [diário de campo: nao quis dar mais detalhes]. (F6)*

*Através de uma menina que trabalha aqui descobrimos o doutor. Descobrimos o número, ligamos, e conseguimos uma consulta particular e depois ele nos transferiu para cá. (P9)*

As perdas no processo de paciéncia-resiliéncia no percurso

Com a demora no aguardo da programado cirúrgica apreendeu-se a "perda do processo de paciéncia- resiliéncia”. A depoente F6 refere-se a espera e ao papel da APS.

*Veio muito decaída e estava muito ansiosa para fazer imediatamente, todo dia que cancelasse era um século. Foi cancelada tres vezes: a primeira por falta do sangue que é O -, a segunda por falta de UTI e a terceira o doutor passou mal. Quando foi a quarta tentativa que deu certo. Desmarcavam ela perdia a paciencia, ficava decaída. (F4)*

*Nao foi muito facinho nao e ainda nao está sendo! Voce ficar mais de 30 dias com um doente é difícil e como acompanhante [diário de campo: se contém para nao chorar], se tivesse mais um pouquinho de esforgo talvez nós nao teríamos ficado este tanto de tempo [diário de campo: no 23° dia de internado; P10 narrou no corredor durante uma de suas caminhadas que seu “problema anal” “migrou” para a uretra. Sua cirurgia precisou de um urologista; o motivo para a demora de 33 dias]. (F6)*

Aguardar por exames complementares e as abordagens deterioram a paciéncia-resignagáo.

*Primeiro contato diz: “A doenga é uma coisa que tem de ser enfrentada”; verbalizando esperanza e formas de enfrentamento. Segundo contato: Pede para nao ser colocada a bolsa de colostomia. As instalares sao criticadas, chama a atengao para o estado da rampa de acesso ao banheiro. Terceiro contato: Pede para registrar que demora muito. Verbaliza que o médico disse que ainda nao a operou por falta de sangue: “Pensam que sou otária. Estou quase pra perder minha paciencia e mandar... (expressao de baixo calao)!” (diário de campo: realizou anastomose, 23 dias de internagao).*

*A instituido é lugar de espera que dá “informales aos pedamos”, (diário de campo P2)*

*Ele diz ‘Mulher quando eu estou em casa... Eu fico disfargando' e aqui fica num cárcere, fica assim ‘Eu quero é ir mim bora, mim bora!'. (diário de campo F2 e P3)*

Conciliam-se a perda da paciéncia-resiliéncia no pré-operatório: tenesmo retal, hematoquezia, melena, diarreia com muco e constante pressáo classificada como "um vento”, fadiga, constipagáo contornada por medicagóes laxativas e as complicares da infusáo da quimioterapia ("dor nas veias”). Com o retorno do CTI as náuseas e anasarca aglutinam-se, aprofundando a impaciéncia. Massagear o abdomen era indicativo para o afastamento, denotando um momento particular de vivéncia da dor restrita ao binomio.

*Fala baixo. Fazem 18 dias de internagao que visivelmente lhe esgotaram e a medicagao nao consegue controlar sua diarreia, impedindo-a de dormir. Conta sobre suas caminhadas e os minutos na capela. Pós-operatório: Anasarca persiste aliada a caquexia nos membros, entretanto diz estar calma apesar do calor da enfermaria provocar um desconforto visível. No 23° dia de internagao veio a óbito. (diário de campo)*

*Decorridos 11 dias e já foi reabordado tres vezes, percebo-o irritado. Refere plenitude gástrica, seu curativo está molhado apesar de ter sido trocado, seus membros edemaciados. “To com muitos gases! Como vou andar com tudo isso?” Diz para a enfermeira, apontando o suporte de medicagao. (diário de campo P7)*

## Discussao

As narrativas obviamente dependem dos discursos médicos e a vertente biomédica é reverenciada. A medicalizagáo existente em verdades laboratoriais e de exames retroalimentadas em um ciclo que vai de usuários, médicos, meios de comunicagáo, indústria farmacéutica e cientistas^10^^,^^18^ transforma usuários em atores, cuja vida orbitará em torno de redes de assistencia (médica) a saúde^18^. Influindo no percurso de acesso a resistencia a deliberado médica e os medos, fazem com que alguns expressem falta de cuidados preventivos ou mesmo a busca por ajuda apenas nas Unidades de Pronto Atendimento (UPA).

Destarte, a partir dos sintomas buscam validagóes com a familia e níveis de saúde informais e quando nao há sucesso requisitam uma validagáo médica. Subjacente as validagóes sociais demoradas os mesmos sáo despojados do conhecimento sobre seus corpos e após a confirmado diagnóstica o as verdades médicas sáo centrais na construgáo do acesso^18^. Para aqueles que náo residem na capital de um estado de dimensóes notáveis possuir rede de apoio na capital é difícil, considerando-se a peregrinado e a construgáo de redes^5^.

Apreender a demora e termos como “atraso diagnóstico” estratificando intervalos de tempo, é fundamental para o debate. Esmiugar casos individuais tem pouca valia, pois percebe-se que as causas da demora váo de encontro aos contextos sociais e temporais. Existem períodos aceitáveis de espera e outros nem tanto como nos casos de hematoquezia, assim tres conceitos sáo cabíveis: de intervalo, contexto causador e de atraso inaceitavelmente prolongado^19^. O atraso foi imputado por parte dos participantes a uma APS frágil e pouco eficiente sem encaminhamentos ou, preocupantemente, atendimento humanizado, demonstrando o descrédito e suscitando: “rede de contatos”, “fluxo náo oficial” e o “paguei no particular” como em outros estudos^4^^,^^5^.

Interpretar tais achados diligencia um panorama analítico menos unidimensional. Pondera-se que geralmente a atengáo básica brasileira náo apresenta condigóes infraestruturais para garantir a conexáo aos demais níveis RAS. Para evitar a peregrinado^20^ um primeiro ponto a ser solucionado é dar reforgo as regióes de saúde, plano de carreira profissional e dedicagáo exclusiva neste nível. Outro ditame económico a ser considerado na realidade brasileira, é a crescente pressáo orgamentária da atengáo hospitalar e especializada afetando os repasses para a atengáo municipal, ao contrário da experiencia de outros países em relagáo a APS.

Este processo resvala no subsentido paguei no particular dando conta que por terem mobilizado recursos financeiros próprios, decerto náo vinculam a saúde suplementar ou privada ao sistema público do qual a totalidade afirmou fazer uso. A expansáo dos planos particulares no país é estimulada pelo fluxo crescente de pacientes do privado para o servigo público, fragmenta e concentra para os níveis genuinamente públicos procedimentos menos rentáveis e onerosos, considera-se que a maior parte dos hospitais brasileiros é privada mesmo que o acesso seja mediado majoritariamente via SUS suplementarmente^21^.

Portanto, um fluxo náo oficial para acessar o sistema público foi desenhado por parcela dos participantes seja da saúde suplementar para o CACON ou mesmo na tentativa de entrada pela APS, depois exames pela saúde suplementar e por fim o CACON. Sendo mediados pela eficiencia de uma rede de contatos fomentadora de apoio social para fins terapeuticos ou curativos: os fluxos náo oficiais desenham-se e variam de acordo com a capacidade de cada indivíduo em dispor de bom capital social^5^. Apesar de voltados para a análise de redes rurais alguns trabalhos sociológicos^22^^-^^23^, abordam- no como micro configuragóes infraestruturais e culturais náo distante das concepgóes normativas e estatais, o fracasso de um grupo ou comunidade ou a baixa resiliencia de um sujeito derivam da ausencia de capital social. Nas cidades existe sob a forma de vantagens infraestruturais, profissionais, rede de contatos e conexóes afetivas institucionalizadas.

Conectado a longa sequéncia psicoemocional desgastante a categoría "As perdas no processo de paciéncia-resiliéncia no percurso", foi narrada no aguardo pela estomizagáo. As queixas váo de encontro ao tédio, "uma eterna cobranga dos médicos", informagóes transmitidas e náo assimiladas e "as mesmas coisas sáo ditas" como indícios da perda da paciéncia em uma instituigáo total como Erving Goffman tratava. Conjectura-se que estes sentidos sáo perdidos paulatinamente, porém os adoecidos náo almejam perder a resiliéncia devido ao forte "poder subjetivo" da mesma. Inteira-se segundo definigáo geral da língua, que paciéncia é o tolerar dificuldades com paciéncia ou "aquilo que demora"^24^.

Enquanto resiliéncia orienta-se ao encontro de um estado pessoal e pouco institucional, o predicado que alguns corpos possuem de voltar a forma original após serem submetidos a deformagóes e provagóes, adaptando-se^24^. No presente estudo a esperanga foi um recurso para que a perda da visáo resiliente náo ocorra, associando-se a esperanga individual como em outra pesquisa para adoecidos com CCR^25^.

O conceito sociológico de resiliéncia^26^ é pertinente a arguigáo para perda de paciéncia como o apreendido. Para a sociologia a resiliéncia náo é um atributo "heroico" inerente a determinado indivíduo, é uma agáo processual e a ideia de comunidade é uma constante nesta quebra epistemológica, pois náo a restringe ao despertar de uma faculdade individual e sim aprendizagem, gestáo de ferramentas para recuperagáo e modos de conviver diante de processos de estresse social, desemprego e dependéncia prolongada de servigos públicos.

Os problemas da construgáo heroica - traumas absorvidos individualmente-familiarmente e solugóes criativas sáo engendradas; tangem a predilegáo em construí-la como um recurso a pobreza e o etnocentrismo, frequentemente denotará as formas de enxergar a vida e solucionar problemas do próprio pesquisador, náo ponderando que adaptagóes sempre ocorreráo enquanto os adoecidos estiverem vivos. Para as políticas públicas explorar o conceito heroico é negativo, leva ao aproveitamento de um recurso oculto, individual e grátis, sem averiguarem a autenticidade da socializagáo de determinantes em saúde, implicando em naturalizar tal característica para qualquer sujeito. O onus é repassado para os sujeitos e náo para a intervengáo pública^26^.

Tais percas comegam na gestáo em saúde e macro política: subfinanciamento, náo dimensionamento de algumas unidades para demanda espontanea, exclusividade para os agendamentos e a prática medica como o única responsável pelo acesso ao SUS^21^^,^^27^. A totalidade destas vicissitudes conhecidas pelo senso comum ativam os recursos ligados a resiliéncia coletiva (atores sociais amizades-parentes^26^. Emprega-se o termo senso comum como sistema cultural, pois para os depoentes ligar a APS a um perfil desfavorável é uma interpretagáo da realidade imediata.

Apenas os recursos externos estressantes náo sáo suficientes para explicar o processo de perca de paciéncia, trés fatores intrínsecos foram apreendidos consistentemente: Fadiga fisiológica, a Dor e Semi-reclusáo em uma instituigáo total. A fadiga oncológica intensifica-se com a quimioterapia, a fraqueza muscular periférica e respiratória, caquexia, deterioragáo do sono, repouso e disfungáo do sistema nervoso^28^, pausando diálogos e suscitando aproximagóes sensíveis. Mesmo sem conclusóes decisivas recente revisáo sistemática^29^ aponta que os sobreviventes ao CCR devem ser estimulados após o tratamento a prática de atividades físicas com ou sem fadiga.

Outro elemento desafiador para a manutengáo da paciéncia sáo as dores, com um forte condutor psicoemocional e comportamental, no qual influencias sociais-culturais e crengas espirituais e religiosas unem-se para administra-la em um ritual privado em alguns casos ou pedindo para que o pesquisador a testemunhasse como objeto concreto. A dor no cáncer avanzado segundo revisáo sistemática com metanálise^30^ mesmo após o tratamento cirúrgico ou antineoplásico, será sentida na maioria dos casos.

Assevera-se que as institutes totais provocam uma crescente ansiedade e intengáo de fuga desta subrealidade incompatível com a célula fundamental da sociedade - a Familia, aspiram sair o mais rápido possível. A permanencia rápida ou náo em institutes totais interfere na "carreira moral” de seus residentes, podendo ser duradoura o suficiente para levar ao receio de enfrentar a vida fora das mesmas^11^ - um exemplo apreendido a nível relacional é o medo de que ninguém cuide adequadamente da estomia após a alta, condicionando que alguns técnicos de enfermagem disponibilizem seus contatos pessoais a fim de instruir telefonicamente os adoecidos.

Desta maneira, pondera-se que o presente estudo tem como limitagóes: a sua composigáo de amostra específica influenciando na apreensáo de resultados do plano de fundo sociocultural desta localidade, contato único no ambulatório antineoplásico e, pela exclusáo dos pacientes que por motivos náo informados, foram encaminhados ao Centro Cirúrgico e náo realizaram o procedimento, o que ofereceria um panorama analítico mais abrangente sobre o acesso. Seguidamente na perspectiva de análise de redes de atengáo, elucubra-se acerca de possíveis vieses decorrentes do nível de atengáo no qual foi realizada a coleta (o terciário), tais como sentimento de medo e ansiedade por conta da programagáo cirúrgica influenciando nas narrativas.

Em suma, a etnografia desvelou aspectos sociais importantes como a medicalizagáo, mobilizagáo em rede concernente ao capital social para acesso verificando ainda o pagamento de exames particulares para obtengáo de tramite mais rápido, a resiliencia heroica e social como recursos durante a permanencia após fatores deletérios internos e externos ao CACON.

## Conclusao

O acesso de pessoas adoecidas por CCR aguardando pela estomizagáo no nível terciário do SUS, indica dependencia dos discursos médicos, demora no trámite entre níveis para a confirmagáo diagnóstica, o "paguei no particular” e percurso desgastante para adoecidos residentes e outras localidades, verificando-se a pronta necessidade de capital social para adquirir consciencia das formas para adentrar a rede e disposigáo para trilhá-la. Isto coloca em descrédito sobretudo a APS e fortalece a compreensáo social de que a saúde suplementar e as operadoras de planos particulares, prestam um atendimento ágil.

Seguidamente, a demora na confirmagáo diagnóstica e sintomas graves sáo fatores estressantes na espera e naturalmente incrementam o desgaste psicoemocional desde a APS e desta forma a resiliencia coletiva precisou ser ativada em um processo de transmutagáo (paciencia-resiliencia) que ocorreu na instituigáo total a partir da fraternizagáo, com a convivencia em espagos de uso comum, contando com o apego a rede de apoio.

Reflexionou-se como uma fortaleza do estudo, que a etnografia contemporánea de ámbito hospitalar preve interagóes proporcionais ao tempo de internagáo da pessoa. A pesquisa contribui para profissionais da assistencia e gestáo de servigos de saúde, por proporcionar conhecer as experiencias de acesso e aguardo pelo procedimento cirúrgico. Futuramente pesquisas devem ser realizadas acerca das redes de resiliencia coletiva de adoecidos com CCR e familiares no meio urbano-rural e no tocante as formas disponíveis para agilizar as vias de acesso dos usuários oncológicos na rede do SUS.
